# The mechanisms of NLRP3 inflammasome/pyroptosis activation and their role in diabetic retinopathy

**DOI:** 10.3389/fimmu.2023.1151185

**Published:** 2023-04-25

**Authors:** Xiaoqin Zheng, Jia Wan, Gang Tan

**Affiliations:** ^1^ Department of Ophthalmology, The First Affiliated Hospital, Hengyang Medical School, University of South China, Hengyang, Hunan, China; ^2^ Department of Orthopedics, The Second Xiangya Hospital, Central South University, Changsha, Hunan, China

**Keywords:** diabetic retinopathy, inflammation, NLRP3, pyroptosis, treatments

## Abstract

In the working-age population worldwide, diabetic retinopathy (DR), a prevalent complication of diabetes, is the main cause of vision impairment. Chronic low-grade inflammation plays an essential role in DR development. Recently, concerning the pathogenesis of DR, the Nod-Like Receptor Family Pyrin Domain Containing 3 (NLRP3) inflammasome in retinal cells has been determined as a causal factor. In the diabetic eye, the NLRP3 inflammasome is activated by several pathways (such as ROS and ATP). The activation of NPRP3 leads to the secretion of inflammatory cytokines interleukin-1β (IL-1β) and interleukin-18 (IL-18), and leads to pyroptosis, a rapid inflammatory form of lytic programmed cell death (PCD). Cells that undergo pyroptosis swell and rapture, releasing more inflammatory factors and accelerating DR progression. This review focuses on the mechanisms that activate NLRP3 inflammasome and pyroptosis leading to DR. The present research highlighted some inhibitors of NLRP3/pyroptosis pathways and novel therapeutic measures concerning DR treatment.

## 1 Introduction

Diabetic retinopathy (DR), a prevalent diabetes mellitus-related complication, is developed by complex pathophysiological mechanisms triggered by hyperglycemia. The early stages of DR are known as non-proliferative diabetic retinopathy (NPDR) and are characterized by increased vascular permeability, retinal hemorrhage and edema, and the formation of microaneurysms (1). NPDR progresses to a more severe stage of the disease, called proliferative diabetic retinopathy (PDR), which is characterized by the formation of pathological retinal neovascularization and eventually lead to retinal detachment and severely compromise vision (2). In the past, DR has been considered a vascular lesion, but in recent years it has been found that retinal nerve cell dysfunction has been observed prior to retinal vasculopathy, so DR is now considered as a neurovascular lesion (3). There is growing evidence that inflammation is a key player in DR, as high glucose-induced production of advanced glycosylated substances, oxidative stress and vascular endothelial growth factor (VEGF) all contribute to the inflammatory response (4), and chronic low-grade inflammation is detected in all stages of DR (5, 6). One of the most studied is the Nod-Like Receptor Family Pyrin Domain Containing 3 (NLRP3) inflammasome (5, 7), the activation of which not only induces the release of inflammatory cytokines interleukin-18 (IL-18) and interleukin-1β (IL-1β), but also pyroptosis, which releases large amounts of inflammatory cytokines, inducing inflammatory cell death in various retinal cells and accelerating the progression of DR (8–11). Retinal cell death is an essential feature of DR (12). However, most previous studies have focused on apoptosis, necrosis, and autophagy. Therefore, in this review, we focus on the activation mechanism of NLRP3 inflammasome/pyroptosis and its significance in DR, and some certain inhibitors of NLRP3 and pyroptosis were examined concerning their possible therapeutic effect on DR.

## 2 NLRP3: a star target in the field of inflammation

The NLRP3 inflammasome is a cytoplasmic immune factor that responds to cellular stress signals and constitutes a sensor (NLRP3), an adapter protein (ASC), and an effector (caspase-1). The ASC protein has a PYD as well as a caspase recruitment domain (CARD). In contrast the NLRP3 is a tripartite protein that consists of a central NACHT domain, a carboxy-terminal leucine-rich repeat (LRR) domain, and amino-terminal pyrin domain (PYD) (13). NLRP3 inflammasomes are typically activated by PAMP (such as microbial toxins, viral RNA, and surface components of bacteria) and DAMP (including ATP, uric acid crystals, beta-amyloid peptides, and aluminum adjuvants) (14, 15). NLRP3 interacts with the homotypic NACHT structural domain upon stimuli to undergo self-oligomerization with the oligomerized NLRP3 causing the bound ASC, attracted through homotypic PYD-PYD interactions, to aggregate into ASC specks (macromolecular focal points) (16–18). Afterward, the assembled ASC recruit caspase-1 *via* homotypic CARD-CARD interactions to generate the NLRP3-ASC-caspase-1 protein complex, called NLRP3 inflammasome (19). The activated NLRP3 inflammasome triggers self-cleavage and activates pro-caspase-1, causing the release of the pro-inflammatory cytokines IL-1β/18. Caspase-1 also cleaves gasdermin D (GSDMD) into N-terminus GSDMD (N-GSDMD), which causes the formation of pores in the cell membrane and triggers pyroptosis, releasing inflammatory factors IL-1β/18 to further promote the inflammatory response (20). Numerous ocular tissues and cells express NLRP3 inflammasome, which is elevated in various ocular disorders (21, 22).

### 2.1 Priming the NLRP3 inflammasome

**Figure 1:**
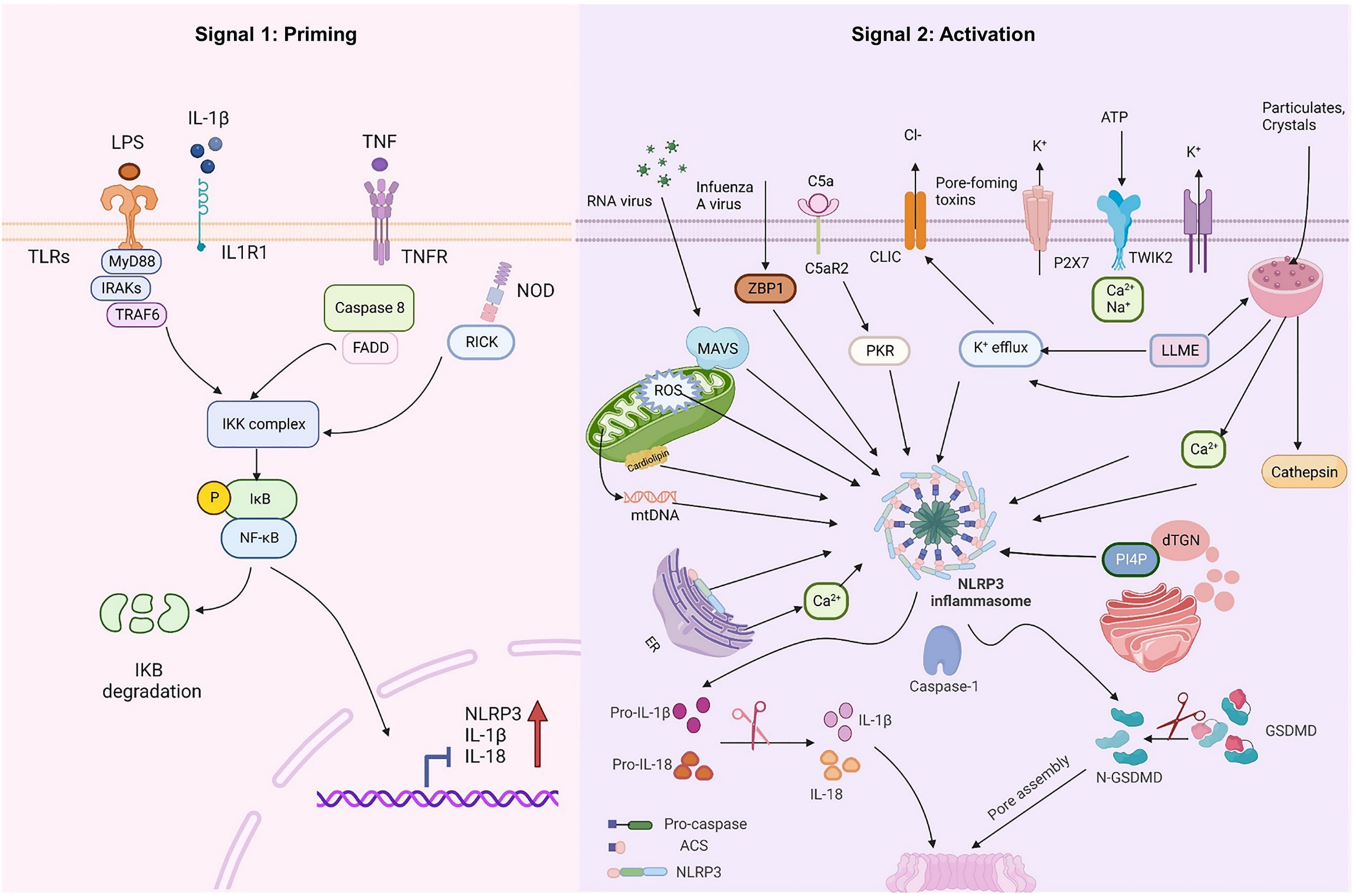
NLRP3 inflammasome priming and activation. The signal 1 (priming, left) is induced by toll-like receptors (TLRs), nucleotide-binding oligomeric structural domain protein (NOD) and tumor necrosis factor receptors (TNFR), which could recognize pathogen-associated molecular patterns (PAMPs) or damage-associated molecular patterns (DAMPs) and upregulate the transcriptional levels of NOD-like receptor thermal protein domain associated protein 3 (NLRP3), proinflammatory cytokines interleukin 1β (IL-1β) and interleukin 18 (IL-18) *via* the myd88-NF-κB pathway. Signal 2 (activation, right) is induced by various PAMPs or DAMPs, such as particulates, crystals, adenosine triphosphate (ATP), K^+^ and Cl^-^ efflux, the disruption of lysosomal, the dysfunction of mitochondrial and the production of mitochondrial reactive oxygen species (mtROS) and mitochondrial DNA (mtDNA), NLRP3 is also activated *via* RNA viruses *via* mitochondrial antiviral signaling protein (MAVS). Formation of the NLRP3 inflammasome activates caspase 1, which subsequently cleaves pro-IL-1β/IL-18 to IL-1β/18. In addition, GSDMD is also cleaved by caspase-1 and inserts into the membrane, causing pores and pyroptosis. *MyD88* myeloiddifferentiationfactor88, *IRAKs* interleukin-1 receptor-associated kinases, *TRAF6* TNF Receptor Associated Factor 6, *FADD* Fas-associated with death domain protein, *IKK* inhibitor of Kappa B Kinase, *IKB* inhibitor kappa B. Created with BioRender.com.

At rest, the expression of NLRP3 and IL-1β in macrophages is minimal and cannot be used to assemble or activate NLRP3 inflammasomes, so their activation must be highly regulated. With few exceptions (23, 24), two steps constitute the NLRP3 inflammasome activation (**Figure 1**): priming (Signal 1) and NLRP3 inflammasome assembly (Signal 2). The priming process has two known functions. The first is to elevate the transcriptional levels of NLRP3 and pro-1L-1β/IL-18. This transcriptional upregulation is triggered by granulocyte-macrophage colony-stimulating factor (GM-CSF) receptors, nucleotide-binding oligomeric structural domain protein 2 (NOD2), TNF receptors TNFR1 and TNFR2, and toll-like receptors (TLRs), which could recognize PAMPs or DAMPs and activate the nuclear factor-κB (NF-κB) (25–27). The second function of priming is to induce post-translational modifications (PTM) of NLRP3, including phosphorylation, ubiquitination, and SUMOylation (28–30), which stabilizes NLRP3 in a signal-competent but auto-suppressed inactive state (14, 23).

### 2.2 Signals for activation of the NLRP3 inflammasomes

After the inflammasome receives the priming signal, the next step involves the recognition of agonists and assembling and activating the NLRP3 inflammasome. While most pattern recognition receptors (PRRs) can only be activated in response to one or a few structurally similar PAMPs or DAMPs, the activation of NLRP3 can be induced under multiple unrelated stimuli, including viral, bacterial, and fungal infections, as well as exposure to environmental irritants and endogenous DAMP-mediated sterile inflammation. These factors are unified in terms of causing cellular stress, which is sensed by NLRP3. Various molecular and cellular processes have been suggested as upstream signals for inflammasome assembly and activation, that include ion fluxes (e.g., Ca^2+^ mobilization, K^+^ and Cl^-^ efflux), lysosomal disruption, mitochondrial dysfunction, reactive oxygen species (ROS), and mitochondrial DNA (mtDNA) release, metabolic changes and dispersed trans-Golgi (31–36). Recent research has indicated that the NLRP3 inflammasome can also be activated *via* various mechanisms including the complement system, protein kinase R (PKR) (37, 38), purine receptor (39), necroptotic signaling, and Z-DNA-binding protein 1 (ZBP1) (40–42). Numerous diabetes-linked metabolic factors act as the secondary signals for NLRP3 activation in DR. These signals include adenosine triphosphate (ATP), cholesterol levels, and cellular structural instability, such as lysosomal rupture, dysfunction of the mitochondrial, and molecular or ionic perturbations including K efflux, ROS, and Ca^2+^ signaling (43). Despite multiple upstream activation events, several pathways are interrelated and overlapped, with ambivalence between the data. Therefore, a consistent model is still unavailable for the activation of NLRP3.

i) Ion flux pathway

K efflux is considered a general upstream event in the activation of the NLRP3 inflammasome. It was observed during the activation of most NLRP3 inflammasomes, except for peptidoglycan, imiquimod, and the related molecule CL097, which can be independent of K efflux (44, 45). However, different activators induced K efflux by diverse pathways. For example, ATP causes K efflux by opening cation channels. Moreover, IL-1β maturation is promoted through K efflux by the purinergic receptor family’s ion channel known as ATP gating of the P2X7 receptor (P2X7R) (46). A recent study has revealed that P2X7 does not function as a cation channel for K efflux even though P2X family receptors are membrane nonselective ion channels for Ca^2+^, Na^+^ and K^+^ (47). P2X7 stimulates the influx of Ca^2+^ and Na^+^ after ATP activation and co-ordinates with the K^+^ efflux-mediated channel tandem pore domain in weak inward rectifying K channel 2 (TWIK2) (48). Subsequently, it induces the binding of downstream NIMA-related kinases (NEK7) to NLRP3, triggering the NLRP3 inflammasome activation (48). A *Streptomyces hygroscopicus-*derived antibiotic, nigericin, serves as a K^+^/H^+^ antiport ionophore that controls the membrane exchange of K^+^ for H^+^ across most membranes (49). Moreover, the complement membrane attack complexes or pore-forming microbial toxins induced damage to the cell membrane integrity can also directly result in K efflux (50, 51). Besides ATP and pore-forming toxins, some particles such as cholesterol crystals, calcium pyrophosphate crystals, and silica also trigger K efflux, essential for activating the NLRP3 inflammasome (31).

For calcium signaling, releasing endoplasmic reticulum (ER)-linked intracellular Ca^2+^ stores or opening plasma membrane channels to allow Ca^2+^ fluxes into the cytoplasm facilitates the NLRP3 complex formation. Moreover, calcium flux and K efflux are usually coordinated when activating the NLRP3 inflammasome. For instance, ATP induces weak Ca^2+^ inward flow and coordinates K^+^ outward flow *via* its receptor P2X7 (48). Subsequently, the release of ER-linked Ca^2+^ is promoted by K efflux (32, 52). The activation of NLRP3 promoted by monosodium urate crystals, alum, nigericin, and membrane attack complexes is dependent on K^+^ efflux and Ca^2+^ flux (3, 53). Additionally, aside from the K^+^ and Ca^2+^, the Cl^-^ channels are also implicated in activating the NLRP3 inflammasome. The most convincing data is that a class of non-steroidal anti-inflammatory drugs (NSAIDs) prevents Cl^-^ migration *via* inhibiting NLRP3 activation by blocking the volume-regulated anion channel (VRAC) on the plasma membrane (54). Similarly, the translocation of Cl^-^ intracellular channel proteins CLIC1 and CLIC4 to the plasma membrane occurs where they mediate Cl^-^ efflux and participate in NLRP3 inflammasome activation (55, 56). In summary, activating the NLRP3 inflammasome is a complex process resulting from a combination of cellular and molecular effects, with many influencing factors. Changes in the concentration of either ion may affect the activation process, and more research is required to understand precisely how ion movement coordinates with NLRP3 activation.

ii) Mitochondrial dysfunction

Mitochondria is emerging as the focal organelle for the activation of the NLRP3 inflammasome, acting as a docking site for its assembly, danger signal release, mtROS production, etc. (57–59). In the resting state, NLRP3 is localized in the ER, and ASC is dispersed in the cytoplasm. During particle stimulation or activation of the inflammasome by the ion carrier Nigerian bacteriocin, acetylated α-microtubule promotes microtubule-dependent mitochondrial translocation to the ER, which can contribute to inflammasome activation in conjunction with ASC on mitochondria and NLRP3 on the ER (35, 60). At least three proteins are thought to act as linkages between NLRP3 and mitochondria: mitochondrial antiviral signaling protein (MAVS), cardiolipin, and mitofusin 2. An inner membrane phospholipid of the mitochondria, the cardiolipin is exposed to the outer membrane in response to mitochondrial stress and functions as a binding site for the molecules linked with autophagy and apoptosis (61). Additionally, cardiolipin independently binds to caspase-1 and NLRP3, and these associations could activate the inflammasome (57, 59). The second protein, MAVS, is articulatory in the RNA-sensing pathway that is essential in triggering the NLRP3 inflammasome during RNA virus infection and following synthetic RNA polyinosinic-polycytidylic acid stimulation (62, 63). It recruits NLRP3, directing its position towards mitochondria for inflammasome activation (64). Finally, mitofusin 2, found at the ER and MAM contact sites, forms a complex with MAVS during RNA virus infection and contributes to the localization of NLRP3 in mitochondria (65).

Besides, ROS is released in mitochondria continuously because of the by-product of oxidative phosphorylation. Even though mtROS level increases during cellular oxidative stress, the damaged or dysfunctioning mitochondria removal can be achieved through mitochondrial autophagy, thus attenuating mtROS production. Inhibiting mitochondrial autophagy or excessive mitochondrial damage increases activation of the NLRP3 inflammasome (35), indicating the participation of autophagy in the regulation of NLRP3. Imiquimod and related compound CL097 target mitochondria to produce mtROS, activating the NLRP3 inflammasome independent of K efflux (44). The activation of the NLRP3 inflammasome signaling in macrophages by phosphatidylcholine oxidation under cellular stress and injury occurs *via* mtROS downstream of intracellular Ca^2+^ signaling (66). Besides the mtROS release and dysfunctioning of mitochondrial, mtDNA could also serve as DAMPs and activate NLRP3 (67–69). Collectively, mtROS and Ca^2+^ could open mitochondrial permeability transition (MPT) pores during oxidative stress (70). The cytoplasmic release of mtDNA depends on the MPT pore and mtROS (67). Upon stimulation by various NLRP3 inflammasome activators, the cytoplasmic release of mtDNA occurs rapidly and is subsequently oxidized. The oxidized mtDNA can then immunoprecipitate with NLRP3 inflammasomes, thereby activating them. In contrast, non-oxidized mtDNA preferentially stimulates the AIM2 inflammasome (69).

iii) Lysosomal disruption

Lysosomal damage is an important factor in activating the NLRP3 inflammasome, which is closely associated to cellular phagocytosis of granules. Endogenous particles (including cholesterol, Monosodium Urate (MSU) crystals, or amyloid β aggregates and deoxygenated sphingolipid-based lipid crystals) or exogenous particulate matter (e.g., alum, silica, and asbestos) are phagocytosed by the lysosomes and accumulated in the lysosomal lumen, leading to greater acidification and swelling of the lysosome and loss of integrity of the lysosomal membrane and release of cathepsin, thus leading to the activation of NLRP3 inflammasome (71). However, the activation of NLRP3 is not activated by a certain cathepsin, as this pathway is only activated in the absence of multiple cathepsins (72). A soluble enzyme regulator, the Leu-Leu-O-methyl ester (LLME), causes the activation of NLRP3 inflammasome through induced lysosomal rupture and increased K efflux (31, 71). It has also been shown that the Ca^2+^-CaMKII-TAK1-JNK pathway promotes the oligomerization of ASC and regulates the activation of NLRP3 inflammasome during lysosome rupture (73). These studies highlight a synergistic effect of lysosomes and ion flow channels in activating the NLRP3 inflammasome.

iv) Trans-Golgi disassembly

The Golgi apparatus and its lipid mediators play a crucial role in activating the NLRP3 inflammasome. NLRP3 stimuli enhance the trans-Golgi network disassembly into vesicles, known as dispersed trans-Golgi network (dTGN), in cell reconstruction systems. The subsequent recruitment and aggregation of NLRP3 are mediated by the phosphatidylinositol-4-phosphate (PI4P) on the dTGN, which is necessary for downstream oligomerization of ASC and activation of caspase-1 (74). Even though the K efflux-independent stimuli (imiquimod, target mitochondria) and K efflux-dependent stimuli (Nigerian bacteriocin) both form dTGN and recruit NLRP3, K efflux is just required for the latter function. This observation indicates the distinct *convergence* of the K efflux- and mitochondria-dependent activation of NLRP3 on Golgi disassembly (74). Research indicated that NLRP3 inflammasome activation depends on sterol regulatory element binding protein 2 (SREBP2) and SREBP cleavage-activating protein (SCAP). SCAP-SREBP2 forms a ternary complex with NLRP3, which translocates into the mitochondria adjacent Golgi apparatus for optimal inflammasome assembly (75). Therefore, NLRP3 activators can trigger various molecular and cellular events, such as mitochondrial dysfunction, ion flux, and lysosomal leakage. Nonetheless, the function of these processes in NLRP3 activation remains largely unclear.

## 3 Pyroptosis

Inflammasome activation triggers the release and maturation of IL-1β/18 and induces an inflammatory programmed cell death (PCD) termed pyroptosis. Pyroptosis is triggered by pathogenic invasion and has been determined to be a CASP activation-dependent process (76–78), hallmarked by the swelling of cells, formation of pores in the plasma membrane, rupture of membrane, and the secretion of pro-inflammatory cytoplasmic contents into the extracellular space, thereby activating a strong inflammatory response (79, 80). Pyroptosis is primarily observed in bone marrow-derived phagocytes, such as neutrophils, macrophages, and dendritic cells. Additionally, keratin-forming cells, endothelium, epithelium, CD4^+^ T lymphocytes, and neurons can also undergo pyroptosis (77). Cells that undergo pyroptosis in the ocular structure include astrocytes, endothelial cells, Müller cells, microglia, retinal ganglion cells (RGCs), pericytes, retinal pigment epithelial cells (RPEs), and corneal epithelial cells (CECs) (81–83). Research has recently depicted GSDMD as the executor regulating pyroptosis (84), with an N-terminal cell death domain (GSDMD^N-term^), a short central junction region, and a C-terminal self-inhibitory structural domain. Activated CASP cleaves the GSDMD at specific protein sites and produces GSDMD-N-terminal and GSDMD-C-terminal. After binding to membrane phosphatidylinositol, phosphatidylserine, and cardiolipin, the N-terminal of GSDMD oligomerizes and inserts itself into the plasma membrane, resulting in the formation of pores, which leads to the release of inflammatory molecules and ultimately triggers pyroptosis (85–88).

### 3.1 Morphological and biological characteristics of pyroptosis

While pyroptosis is similar to apoptosis and necroptosis, it has several distinguishing features in contrast with other types of PCD. During pyroptosis, cells undergo chromatin condensation and DNA breakage, while the nucleus remains intact, the cell swells, the plasma membrane ruptures, and inflammatory cytokines are released (**Table 1**) **(** 89). In contrast, apoptosis caused by CASP activation results in membrane blebbing and nuclear fragmentation, while the plasma membrane is left intact and does not cause an inflammatory response *in vivo* (89). Additionally, no chromatin condensation or loss of plasma membrane integrity occurs in ferroptosis but causes mitochondrial condensation, reduction or loss of mitochondria cristae, and increased membrane density (90). On the other hand, chromatin condensation does not occur in cells undergoing necroptosis; however, their nuclear membranes are ruptured (91). Like GSDMD in pyroptosis, MLKL oligomers act as executive proteins to mediate cell death during necroptosis (92). In contrast, unlike pyroptosis, necroptosis is independent of CASP but requires RIPK3-regulated phosphorylation of MLKL. The phosphorylated MLKL generates a pore complex at the plasma membrane, leading to the secretion of DAMP, cell swelling, and membrane rupturing (93). MLKL forms selective channels in the plasma membrane that induce an influx of ions, increasing intracellular osmotic pressure, inward water flow, and severe cell swelling. However, pyroptosis depends on CASP to cleave the GSDMD directly and form pores. The channels are not selective, and the intracellular and extracellular ionic osmotic pressure gradient disappears. However, the intracellular colloidal osmotic pressure is higher, and water flows inward, leading to increased cell size and subsequent cell lysis, but the swelling is less severe than in necroptosis (85, 89, 94, 95). NLRP3 is a major contributor to the pyroptosis but not to other cell death modes (apoptosis, necroptosis, ferroptosis and autophagy, *etc.*), but signals (K^+^ and mtDNA, *etc.*) associated with the release of other cell death can activate NLRP3, thereby inducing inflammation or pyroptosis (96, 97). In fact, various cell death modes can crosstalk each other through NLRP3.

**Table 1:** The comparison of different modes of cell death.

Types	Pyroptosis	Apoptosis	Necroptosis	Ferroptosis	NETosis	Autophagy	Cuproptosis
**Death stimulus**	DAMPs and PAMPs, dsDNA, pathogens, LPS, ATP, permeability of cell membranes to K^+^	Cell stress; DNA damage, infection, hypoxia, ligands of transmembrane receptor (TNFR1, FAS)	Physical or chemical trauma, Pathogenic infections or Pathological stimuli	Decreased uptake of cysteine or glutamine, increased iron uptake, suppression of GPX4	Various pathogens, such as bacteria, fungi, protozoa, viruses, bacterial cell wall components-LPS	Starvation, hypoxia, oxidative stress, protein aggregation and ER stress	Copper accumulation
**Initiator**	Activation of inflammasomes (e.g., NLRP3, NLRC4, AIM2, pyrin, etc.)	Death receptor (TNF receptor superfamily) activation/mitochondrial events (intracellular signals)	The activation of cell surface death receptors (FasRs, TNFR, IFN, and TLRs), and ZBP1 in cells	The suppression of the cystine–glutamate antiporter (system Xc^−^), depletion of GSH and inactivation of GPX4	Activation of neutrophil, including the activation of surface receptor (TLR2, complement component 3, GPCRs, TNF and Fc receptors)	Serine–threonine protein kinase ULK1 complex (such as ATG13, ATG101 and FIP200)	Binding of Cu^2+^ to lipid acylated components of the TCA
**Executor**	Caspase‐1, caspase‐4/5/11 and GSDMD	Caspase‐3, 6,7 and caspase 8	MLKL oligomerization and translocate to the plasma membrane	Iron-dependent phospholipid peroxidation	Dysregulated NETs, GSDMD mediates membrane pore formation	Autophagosome, autophagolysosome	Loss of iron-sulfur cluster proteins and DLAT Oligomerization
**Morphological characteristics**	Cells swell, cell membranes rupture, organelles are lost, chromatin condense, nuclei remain intact	Cell shrinkage, nuclear fragmentation, chromosomal DNA fragmentation, plasma-membrane blebbing and apoptotic vesicle formation	Cell and organelle swelling; chromatin condensation; cell membrane rupture and release of cytoplasmic contents	Lipid peroxides accumulation, less dense than normal mitochondria, reduced or absent mitochondrial cristae, ruptured outer mitochondrial membrane	Nuclear swelling, dissolution of nuclear and cytoplasmic granule membranes, rupture of the cytoplasm	Large accumulation of bilayer membrane autophagic vesicles (autophagosomes) in the cytoplasm; fusion of autophagosomes with lysosomes to degrade contents	Mitochondrial shrinkage, mitochondrial membrane rupture
**Inflammation**	Yes	No	Yes	Yes	Yes	Partially have	/
**NLRP3 participation**	Yes	No	No	No	No	No	/
**Functions**	Against pathogens invasion and enhance antitumor immunity, neurological related disorders	Maintenance of homeostasis of the internal environment	Defense to pathogens infections and maintain tissue homeostasis	Tumor suppression, inflammation and immune surveillance (Cancer immunity, neuroinflammation)	Involvement in innate immunity, defense against bacterial and fungal infections	Maintaining intracellular homeostasis, excessive autophagy led to metabolic stress	Enhance antitumor immunity

DAMP danger-associated molecular pattern, PAMP pathogen-associated molecular pattern, dsDNA double-stranded DNA, LPS lipopolysaccharide, ATP adenosine triphosphate, TNFR1 tumor necrosis factor receptor 1, ER endoplasmic reticulum, NLRP NOD-like receptor protein, AIM2 absent in melanoma 2, INF interferon, TLR toll-like receptor, ZBP1 Z-DNA binding protein 1, GSH glutathione, GPX4 glutathione peroxidase 4, GPCR G-protein-coupled receptors, ULK1 Unc-51-like kinase, ATG13 Autophagy Related 13, ATG101 Autophagy Related 101, FIP200 focal adhesion kinase family interacting protein of 200 KD, TCA tricarboxylic acid cycle, GSDMD gasdermin D, MLKL mixed lineage kinase domain-like, NETs neutrophil extracellular traps, DLAT Dihydrolipoyl Transacetylase.

### 3.2 Activation mechanisms for pyroptosis

**Figure 2:**
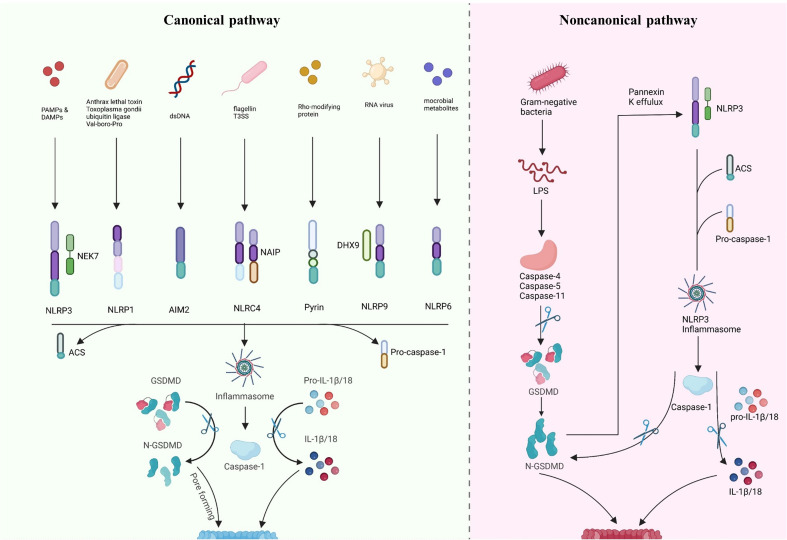
Inflammasome-dependent pathway of pyroptosis. Canonical pathway: inflammasome sensors are activated by different signals, such as dsDNA, flagellin T3SS, Rho-modifying and RNA virus. Activated inflammasome sensors subsequently oligomerization with pro-caspase1 and ASC. Activated caspase1 cleaves pro-IL-1β/18 into mature IL-1β/18. Caspase1 also cleaves GSDMD to release the N-terminal domain (GSDMD-N), which then inserts into the membrane, causes pores and induces pyroptosis. Noncanonical pathway: LPS directly activates caspase-4/5/11, causing GSDMD cleavage and triggering intracellular K efflux, K efflux further activates the NLRP3 pathway and accelerates pyroptosis. Created with BioRender.com.

Gasdermin-mediated pyroptosis includes both inflammasome-independent and-dependent pathways (**Figure 2**). Typically, pyroptosis dependent on inflammasome includes the CASP4/5/11- and CASP1-dependent pathways (non-canonical and canonical, respectively). Recent research has indicated new inflammasome-independent pathways, including the pathways mediated by CASP-3/8 and Granzyme A (GZMA) secreted by cytotoxic lymphocytes, which can sever GSDMB to release the GSDMD-N-terminal fragment, causing cell perforation and inducing other GSDM-mediated pyroptosis (98–100). The canonical pathway is mediated by inflammasome assembly, GSDMD cleavage, and secretion of IL-1β/18. The assembly of inflammasomes begins with PRRs (termed inflammasome sensors), such as NLR, AIM2, and pyrin, which recognize pathogen-associated molecular patterns and risk-associated molecular patterns (PAMP and DAMP). PRRs bind to ASC, a caspase-1 precursor, forming a multi-protein complex and activating caspase-1. The caspase-1, in turn, cleaves GSDMD to create the active domain (GSDMD-N terminal) containing peptide, which causes the cell membrane to become perforated and ultimately ruptures, releasing the contents, leading to an inflammatory reaction (88, 101). Moreover, activated caspase-1 cleaves pro-IL-1β/18, forming extracellularly released activated IL-1β/18, leading to aggregated inflammatory cells and an amplified inflammatory response (20, 84, 86, 102, 103). In the non-canonical pathway, caspase-4/5/11 recognizes intracellular lipopolysaccharide (LPS) directly (104) and cleaves GSDMD, triggering pyroptosis (105). However, caspase-4/5/11 cannot cleave pro-IL-1β/18 but can mediate their maturation and secretion *via* the NLRP3/caspase-1 pathway in partial cells, indicating the significance of caspase-1 in the production of mature IL-1β and IL-18 (106–108). In addition, cleavage of GSDMD by caspase-4/5/11 could trigger intracellular K efflux (20), causing the activation of NLRP3 inflammasome and accelerating pyroptosis (109–111). Further, in response to LPS stimulation, activated CASP11 cleaves Pannexin-1, induces ATP efflux, and binds to P2X7R, triggering NLRP3-linked pyroptosis (102, 112).

## 4 NLRP3 and pyroptosis in diabetic retinopathy

In the working-age population, the most prevalent cause of preventable blindness is diabetic retinopathy, a common and distinct microvascular complication of diabetes (113). The retina is a complex system, usually made up of ten layers (from the inside out): RPE, optic rod and cone layer, and outer membranes consisting of limiting membrane (OLM), nuclear layer (ONL), plexiform layer (OPL), as well as inner membranes such as nuclear layer (INL), plexiform layer (IPL), cell layer (GCL), fiber layer (NFL) and limiting membrane (ILM) (114). In terms of histology, blood cells (endothelial and pericytes), glial cells (astrocytes, Müller cells, and microglia), and retinal neurons form an essential structure called the retinal neurovascular unit (NVU) (115). A growing body of research suggests that the abnormal interactions between inflammation, oxidative stress, mitochondrial dysfunction, advanced glycation end-products (AGEs) and cell death leads to retinal vascular abnormalities, blood-retinal barrier (BRB) disruption and neurological dysfunction (116–118). Recent studies have shown that chronic inflammation plays a crucial role in the pathogenesis of DR, where the NLRP3 inflammasome is of particular significance. Research depicts that peripheral blood mononuclear cells of individuals with DR exhibit greater expression levels of protein and gene of caspase-1, ASC and NLRP3 in contrast with normal individuals. Moreover, increased expression levels of the NLRP3, caspase-1, and the pro-inflammatory factors IL-1β/18 are also evident in the vitreous humor (119). Similarly, patients with proliferative DR show an elevation of ASC and NLRP3 in their fibrovascular membranes (7), while inhibiting NLRP3 inflammasome slows DR progression (120). The activation of NLRP3 can induce an inflammatory response and act as a bridge between neovascularization and the inflammation (121). Most importantly, NLRP3 activation can induce pyroptosis, leading to inflammatory cell death in retinal cells. Cell death is a typical characteristic of DR, which has a major role in the onset and advancement of DR (122). However, a large number of studies in the past have focused on cell death such as apoptosis, autophagy and ferroptosis. Recently, it has been shown that pyroptosis can be observed in neurons, RMEC, Müllers, microglia and RPEs.

### 4.1 The role of NLRP3 inflammasome in diabetic retinopathy

**Figure 3:**
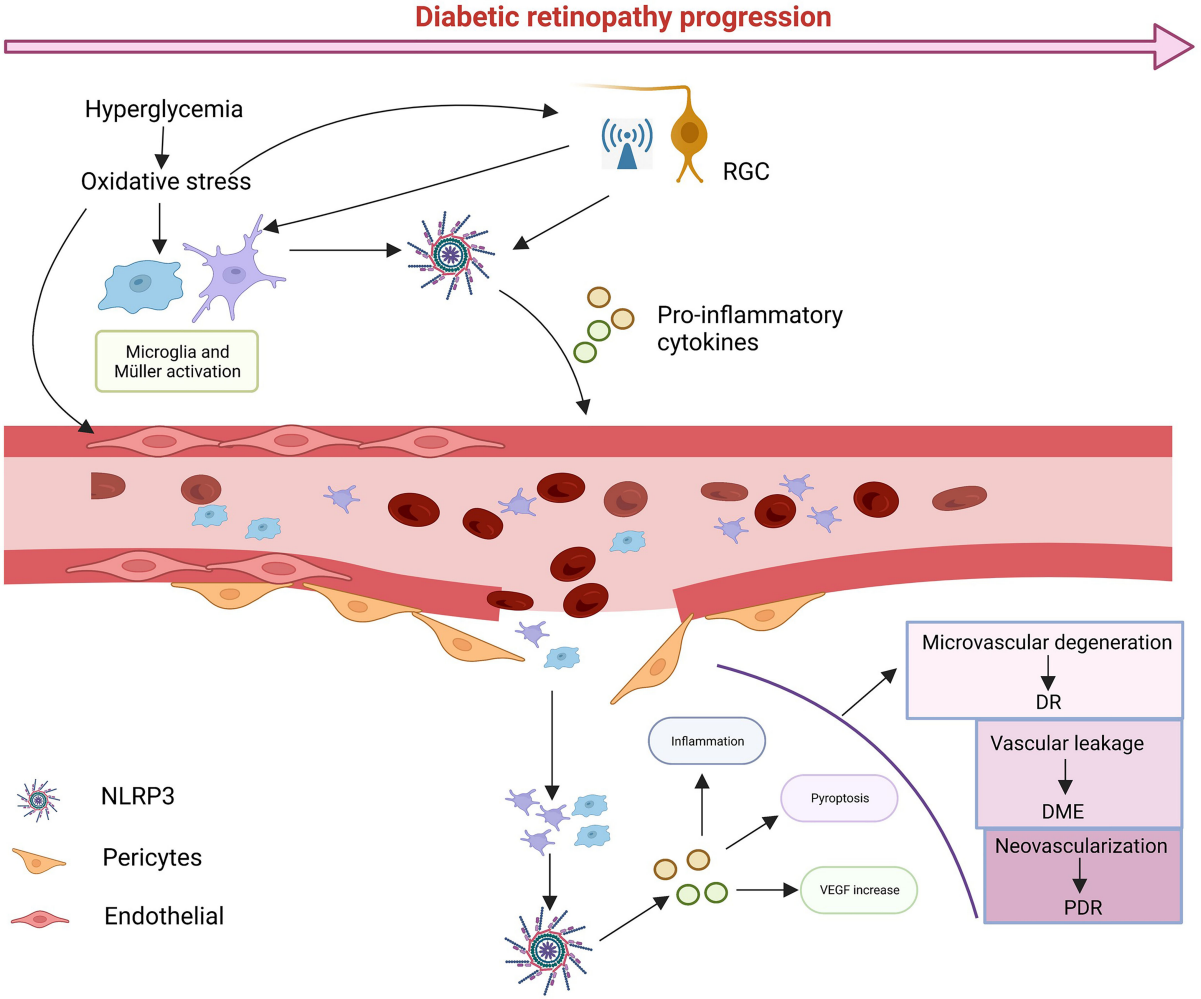
The possible cascade response. In diabetic conditions, glucose-induced metabolic abnormalities and oxidative stress activate NLRP3 in RGCs, leading to high levels of abnormal signaling, while microglia and Müller cells are activated. Sustained glucose further activates NLRP3, which leads to damage to pericytes and vascular endothelial cells, leading blood leakage and creating an ischemic and hypoxic environment, in turn, damaging vascular units. With time, various types of cell death can occur, and ultimately leads to damage to vision. Created with BioRender.com.

When the retina is exposed to glucose stimulation, abnormalities in various metabolic pathways lead to cellular oxidative stress and inflammation, and as signal regulators, RGCs may be the first to be damaged, as it has been shown that NLRP3 activation and electroretinogram defects precede the onset of microvascular lesions (123, 124). With RGCs damage, many abnormal signals are released to stimulate microglia and Müller cells to activate and proliferate (125). At the same time the metabolic abnormalities caused by high glucose also stimulate glial cells to activate NLRP3 inflammasomes, releasing anti-inflammatory cytokines to protect the retina (126, 127). However, continued glucose stimulation leads to excessive production of pro-inflammatory cytokines by glial cells and damage to retinal neurovascular units. As glial cell damage and phagocytosis decline, leading to the accumulation of metabolic wastes such as ATP, ROS, mtDNA or toxic chemicals such as serum uric acid (UA) (128), NLRP3 in the retinal vascular endothelium and pericytes is activated and large amounts of the inflammatory cytokines IL-1β and IL-18 are released (129–131). At this point retinal vascular cells begin to deteriorate, the pericytes are shed and leukocytes and macrophages are induced to aggregate and adhere tightly to vascular endothelial cells with the upregulation of adhesion molecules (132, 133), leading to further destruction of the retinal BRB, which leads to vascular leakage, edema and hypoxia. The hypoxic microenvironment disrupts the balance between angiogenic (*e.g*., VEGF) and anti-angiogenic (*e.g*., PEDF) regulators, promoting neovascularization (121, 134). Activation of NLRP3 induces the releases of IL-1β and IL-18. IL-1β binding to IL-1R increases retinal vascular permeability, exacerbating hypoxia, IL-18 is involved in pro-angiogenesis with VEGF (5). Most importantly, NLRP3 acts as a major contributor to pyroptosis. Numerous studies have shown that almost all retinal cells can undergo pyroptosis and the cells swell and rupture, releasing inflammatory cytokines and triggering an inflammatory storm. With the progress of DR, excessive activation of NLRP3 induces inflammation and various retinal cell death, triggering an inflammatory storm that leads to structural and functional collapse of the NVU and ultimately impairs vision (**Figure 3**).

### 4.2 Mechanism of NLRP3/pyroptosis activation in different cells in diabetic retinopathy

**Figure 4:**
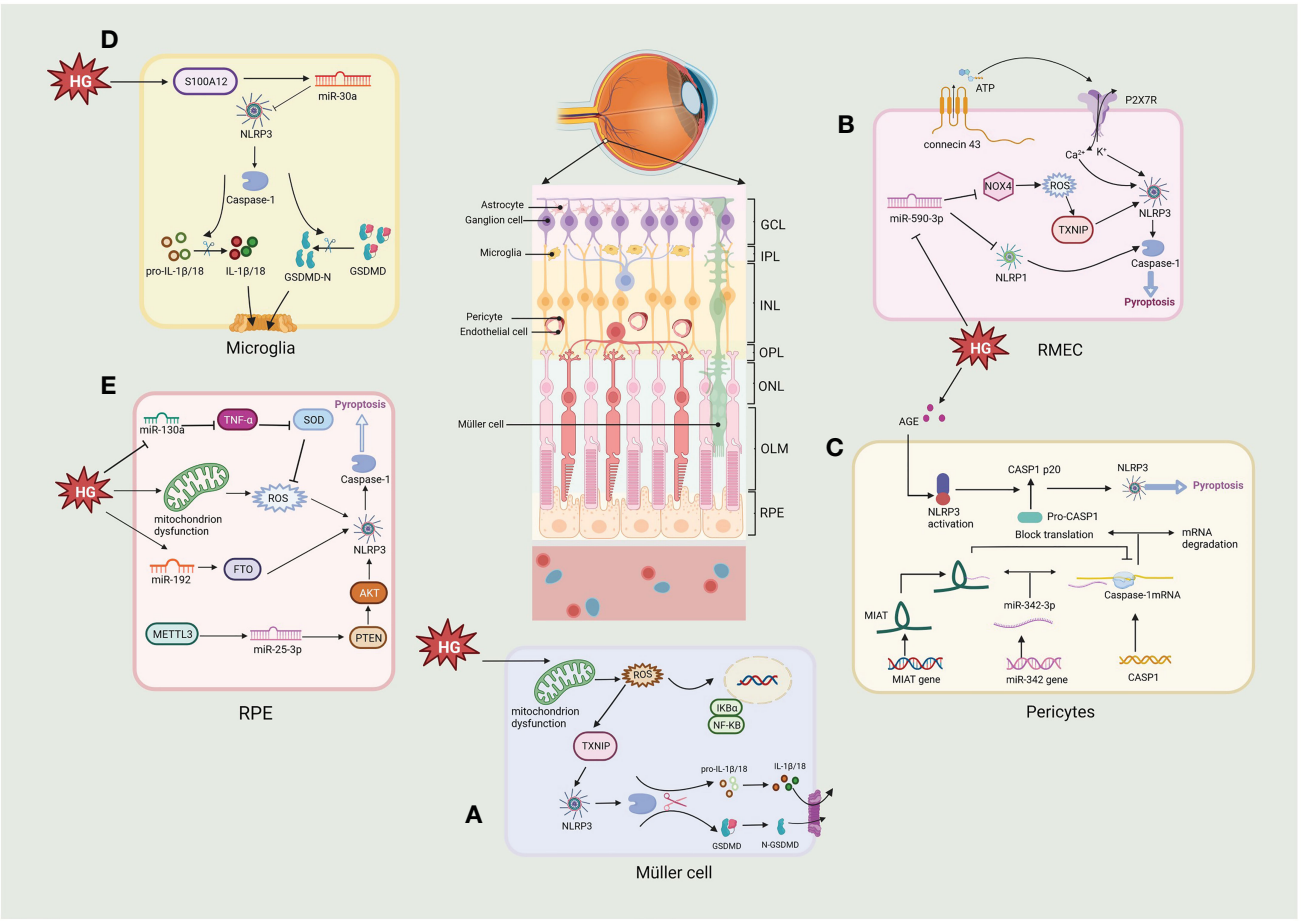
Molecular signaling pathway of NLRP3/pyroptosis in diabetic retinopathy. **(A)** Müller cell: hyperglycemia activates the NLRP3/pyroptosis *via* the ROS/TXNIP pathway. **(B)** Retinal microvascular endothelial cell (RMEC): high glucose induces an increase in extracellular ATP through connexin43 and increased binding to P2X7R, which then causes K^+^ efflux and Ca^2+^ influx by activating the NLRP3 inflammasome. High glucose also activates pyroptosis *via* the miR-590-3p/NOX4/ROS/TXNIP/NLRP3 pathway, with miR-590-3p also targeting NLRP1. **(C)** Pericytes: increasing lncRNA MIAT competes with CASP1 mRNA for binding to miR-342-3p, thereby inhibiting the CASP1 translation and pericytes pyroptosis. **(D)** Microglia: hyperglycemia produces S100 protein (S100A12), which induces the activation of NLRP3 *via* a miR-30a-dependent mechanism. **(E)** Retinal pigment epithelium: hyperglycemia activates the NLRP3/pyroptosis through triggering the generation of mtROS, also *via* miR-130a/TNF-α/SOD1/ROS, miR-192/FTO/NLRP3 or METTL3/miR-25-3p/PTEN/Akt/NLRP3 signaling pathway. Created with BioRender.com.

Retinal pericytes (RPs) and vascular endothelial cells are essential elements of the retinal microvascular system and the internal of BRB (**Figure 4**). The loss of RPs can trigger microaneurysm formation, blood leakage, edema and ischemia, and induce proliferative neovascularization of the retina and subsequent loss of endothelial cells, the death of RPs and endothelial cell is a fatal blow to the NVU and accelerates the progression of DR (135, 136). Several models of DR have depicted the activation of NLRP3/caspase-1 and the release of IL-1β in retinal microvascular endothelial cells (RMECs) and retinal endothelial cells (RECs) *in vitro* and *in vivo* experiments (137–139). Connexin 43 is a cell-cell communication channel (gap junction) forming protein (140, 141), which is expressed in HRMECs, Müller cells, microglia, RPE, and astrocytes in the retina (142). Research demonstrates that Connexin 43 is upregulated in mouse DR models (142). Hemichannels open under hypoxic-ischemic conditions to form membrane pores, resulting in elevated extracellular ATP (143). A non-selective cation channel is formed when increased extracellular ATP links to a NLRP3 inflammasome activator, the P2X7R, a receptor which detects metabolic stimuli and oxidative stress. This results in initiating pyroptosis in human RMEC due to the subsequent K efflux and Ca^2+^ influx activating the NLRP3 inflammasome (144–146). Another study demonstrated that P2X7 was expressed at considerably elevated levels in RMEC in the presence of high glucose and LPS and that the persistent presence of high extracellular ATP could induce the P2X7 macropore opening. Activated P2X7 activates NLRP3 inflammasomes *via* K efflux, ROS, and glutamine efflux, while LPS activates NLRP3 inflammasomes *via* the caspase-1 and 11 (canonical and non-canonical, respectively) pathways to induce pyroptosis (147). Moreover, Yang et al. observed that RMEC exposed to advanced glycation end product modified bovine serum albumin (AGE-BSA) showed features of pyroptosis such as cell swelling and nucleus fragmentation, and the western blot results showed that expression of NLRP3, GSDMD and caspase-1 were upregulated. These phenomena were inhibited by H3 relaxin and MCC950, suggesting that NLRP3 is activated and induces pyroptosis in RMEC under a variety of pathological conditions (148). ROS, a known DAMP, triggers NLRP3 activation in DR (149–151). In endothelial cell, ROS induce pyroptosis by activating the NLRP3 through the initiation and activation process. The initiation phase refers to upregulation the expression of NLRP3, IL-1β/18 and caspase-1 expression by ROS. The activation phase refers to the promotion of NLRP3 inflammasome assembly and activation by ROS *via* TXNIP (152). Chen et al. found that minocycline, a tetracycline antibiotic, significantly downregulated ROS production and deceased TXNIP expression, inhibiting NLRP3 activation and decreasing the secretion of IL-1β/18 (138, 153). In a human RMEC model incubated with high glucose, miR-590-3p was downregulated and promoted pyroptosis by activating the NOX4/ROS/TXNIP/NLRP3 pathway and targeting NLRP1. In addition, increased levels of IL-1β further exacerbated cell death by inducing miR-590-3p downregulation through positive feedback (139), suggesting an involvement of the microRNAs in the onset of NLRP3-mediated pyroptosis.

Retinal pericytes (RPs) modulate the production of tight junction proteins and support the vessel wall structurally (154). Gan et al. showed that glucose induced inflammation, pore formation and pyroptosis in RPs with increasing glucose treatment time and dose. The NLRP3 inhibitor glyburide or the caspase-1 inhibitor YVAD could reverse these phenomena, suggesting that glucose induced pyroptosis *via* NLRP3-caspase1-GSDMD in RPs (130). Another study using AGE-BSA to mimic the DR environment showed increased caspase-1, GSDMD-N, IL-1β/18, and LDH expression in RPs (155). These studies demonstrate that elevated glucose can induce inflammation and pyroptosis of RPs. Furthermore, lncRNA MIAT and CASP1 levels considerably elevated in AGE-BSA-treated RPs, whereas the expression of miR-342-3p was reduced. This observation indicates that CASPA1-dependent pyroptosis of RPs may be promoted due to the overexpressed MIAT which competes with CASP1 for conjugation to miR-342-3p, thus disrupting the inhibitory impact of the miRNA on CASP1 (155). Consequently, the MIAT/miR-342-3p/CASP1 pathway may provide a unique perspective on pericyte loss, contributing to developing novel therapeutic approaches for DR.

The retina’s structural support and nutrient metabolism are associated with Müller cells, the most pre-dominant and extensively dispersed macroglia in the retina (156). Müller cell death promotes loss of BRB integrity, increased vascular permeability and loss of protective effects on neuronal and vascular cells. Loss of Müller cells in diabetes is also associated with aneurysm formation, a clinical feature of DR (156). Whether Müller cells die in DR has been debated, this is because it secretes neurotrophic factors that protect them from hyperglycemia, at least in the beginning of DR. However, recent studies have shown that Müller cells actually begin to die gradually as the course of DR progress and Müller cell death is rapidly accelerated when protective growth factors are diminished (157). Studies have shown that Müller cells died in the DR displayed hypertrophy, a characteristic similar to pyroptosis. However, due to the lack of understanding concerning pyroptosis at the time, it was difficult to distinguish the mode of cell death (158, 159). Further research showed that caspase-1 activation and production of IL-1β were observed in rat retinal Müller cells cultured in a high-glucose environment (160). Moreover, elevated caspase-1, ASC, NLRP3, and IL-1β were also detected in 30mM glucose-treated mouse retinal Müller cells (161). Oxidative stress and the NLRP3 inflammasome are bridged through TXNIP. The expression of TXNIP in Müller cells is upgraded through genomic modifications during chronic hyperglycemia (138, 161, 162). Increased mitochondrial activity in DR produces high ROS as cells attempt to process excess glucose (67, 163). Subsequently, ROS generates oxidized disulfide bonds and releases TXNIP (164, 165); free TXNIP assists NLRP3 assembly with pro-caspase-1 and ASC and activates the NLRP3 inflammasome, thereby activating the pyroptosis (138). In another study it was possible to observe that HG significantly induced the expression of proteins of GSDMD, NLRP3 and caspase-1, after treatment with N-acetylcysteine (NAC), the expression of GSDMD, NLRP3 and caspase-1 were significantly reduced in Müller cells, indicating HG induced pyroptosis in Müller cells *via* NLRP3/pyroptosis (166). These studies indicate that Müller cells undergo pyroptosis in DR.

In the eye, microglia play an important role in immunity, that surveil the peripapillary environment and process the removal of metabolic wastes from the retina (167). Recent studies have shown that hyperglycemia produces S100 protein (S100A12), which induces microglia activation and inflammatory responses by modulating NLRP3 activity. It also stimulates the miR-30a-dependent secretion of IL-1β/18 from microglia (168). High glucose induces a shift in retinal microglia to the M1 phenotype and promotes the release of pro-inflammatory cytokines (e.g., IL-1β), and subsequently Huang et al. found that microglia showed a significant decrease in cell viability under high glucose (25, 50 or 100 mM) conditions and that lactate dehydrogenase (LDH) release and caspase-1 activity increased with increasing glucose concentration. In addition, protein expression of IL-1β, caspase-1, NLRP3 and cleaved GSDMD was increased. However, pretreatment with either the NLRP3 inhibitor MCC950 or the caspase-1 inhibitor Z-YVAD-FMK significantly inhibited pyroptosis under high glucose (25 mM) conditions, and the results adequately demonstrate that glucose induces pyroptosis *via* the NLRP3/caspase1-GSDMD pathway in retinal microglia, releasing more inflammatory factors, exacerbating the inflammatory response in the retina and induce NVU damage in the DR (169). In addition, ischemia and reperfusion (I/R) damage underlies many retinal diseases, such as glaucoma, DR and central retinal artery occlusion (170). retinal I/R have been shown to promote pyroptosis of retinal microglia, associated with increased expression of lncRNA-H19. Increased lncRNA-H19 significantly promotes NLRP3/6 inflammasome imbalance, leading to cytokine overproduction and microglia pyroptosis, while lncRNA H19 knockdown effectively inhibits these effects (171). Another report indicated that NLRC5 directly binds to the NLRP3/NLRC4 inflammasome and synergistically drives microglia pyroptosis (172). The above researches suggest that inflammation and pyroptosis of retinal microglia activated by hyperglycemia might play an essential role in the progression of DR.

RPEs form the external BRB and regulate the structure and functioning of the retinal, RPEs act as a cellular barrier separating the neuronal retina and the fenestrated choriocapillaris, and disruption of the RPE barrier plays a pathogenic role in the development of DR (4). A high glucose environment results in pyroptosis of RPE cells through a series of events, starting with the plasma membrane passage of glucose, which then generates mitochondrial ROS, leading to NLRP3 inflammasome activation, cleavage of CASP1, and IL-1β/18 release (173). It was shown that pyroptosis-linked proteins such as Caspase-1, GSDMD, NLRP3, and IL-1β/18 were upregulated in a high glucose environment. In contrast, methyltransferase-like protein 3 (METTL3) overexpression regulated the miR-25-3p/PTEN/Akt/NLRP3 pathway through a DGCR8-dependent approach to reduce the hyperglycemic-induced pyroptosis in RPEs (8). Gu et al. demonstrated that glucose induced the upregulation of NLRP3, GSDMD, IL-1β/18 and caspase-1 in a time- and dose-dependent manner in RPEs, which was later inhibited by the expression of miR-192. The FTO alpha-ketoglutarate-dependent dioxygenase (FTO), a downstream target of miR-192, increases NLRP3 expression by promoting NLRP3 demethylation, while miR-192 overexpression inhibits high glucose-induced RPEs pyroptosis by negatively modulating the FTO/NLRP3 signaling pathway (174). In another study, high glucose (50 mM) increased the expression levels of caspase-1, GSDMD, NLRP3, IL-18/1β in ARPE-19 cells and induced ROS production in a time-dependent manner. In previous studies it was shown that ROS could activate NLRP3, which in turn activate the pyroptosis. Further results verified that the ROS scavenger NAC and the GSDMD inhibitor necrosulfonamide (NSA) reversed the effect of high glucose on pyroptosis in ARPE-19 cells. Mechanistically glucose significantly reduced miR-130a, which activated NLRP3-mediated pyroptosis *via* the TNF-α/SOD1/ROS axis (175). The redox pathway of Trx is essential for maintaining normal RPE function in the DR. Under hyperglycemia, TXNIP expression is upregulated, leading to cellular oxidative stress, lysosomal dysfunction, and mitochondrial damage. Therefore, Thangal et al. treated RPEs with Auranofin (a TrxR inhibitor), resulting in cellular mitochondrial dysfunction and oxidative stress, along with enhanced activity of the pro-inflammatory caspase-1 in RPEs. These effects of Auranofin on RPEs could be inhibited by the antioxidant NAC, while neither ferrostain-1 no necroptosis-1 (inhibitors of ferroptosis and necroptosis, respectively) did not inhibit this death. In contrast, MCC950 or Ac-YVAD-cmk (Caspase-1 inhibitors) significantly reduced LDH release, suggesting that high glucose-induced oxidative stress promotes cell death *via* NLRP3-caspase1-pyroptosis rather than other cell death modes, providing sufficient evidence that NLRP3 mediates pyroptotic cell death in DR (176). Overall, there is increasing focus concerning the involvement of RPE-related pyroptosis in the DR.

RGCs are the output neurons that integrate data, while retinal neurons are the core cells that transfer optical signals and create vision (177). Several studies have confirmed neuronal changes, even before clinical vascular changes in DR (178, 179). The immunohistochemical analysis of a diabetic rat model depicted that caspase-1, ASC, and NLRP3 were localized primarily in the inner and outer nuclear layers and the ganglion cell layer. Furthermore, the number of cells expressing the abovementioned factors was considerably elevated in diabetic rats and intravitreal injection of drugs that inhibit NLRP3 inflammasome and IL-1β/18 expression (180). High glucose could lead to hypoxia and an imbalance in the immune response of retinal tissues. Under hypoxic conditions, continuous production and degradation of hypoxia-inducible factor-1 (HIF-1) were detected, activating the IL-6 and IL-8 genes by acting as transcription factors. In addition, pericyte loss may lead to cell-free capillary formation, which is linked to vascular occlusion and further leads to retinal non-perfusion and ischemic-hypoxic damage. Ischemia and hypoxia could further upregulate HIF-1 expression. Pyroptosis contributes to retinal ischemia injury and contributes the death of RGCs through the signaling pathway caspase-8-HIF-1α-NLRP12/NLRP3/NLRP4 in acute glaucoma (181). Caspase-mediated pyroptosis can also be observed in RGCs in various retinal diseases (182). For example, in some optic nerve compression damage models, NLRP3 expression is enhanced in retinal microglia, promoting IL-1β and caspase-1, while knockdown of NLRP3 slows RGC reduction after partial optic nerve compression damage (183). Future studies are essential to detect whether all retinal cells undergo pyroptosis in DR.

In summary, elevated intracellular glucose levels in diabetic patients trigger oxidative stress, leading to intracellular ROS and ATP production, and sustained glucose stimulation leads to mitochondrial dysfunction, which induces additional mtROS leakage and mtDNA release, all of which together activate NLRP3. Activation of NLRP3 can be detected at all stages of DR, suggesting that NLRP3 is involved in the development of DR. Firstly activated NLRP3 inflammasome can provide a platform for the maturation of IL-1β/18, thereby inducing inflammation. Most importantly, the NLRP3 is also involved in pyroptosis, inducing inflammatory cell death. Excessive cell death and inflammation leads to BRB rupture, blood infiltration, edema and retinal detachment, ultimately damaging vision. However, almost all cells are capable of pyroptosis in DR, it is still unclear which cells undergo pyroptosis first or how these cells interact during and after pyroptosis further studies are needed. In addition, NLRP3 may also be involved in other modes of cell death, we just focus on the role of pyroptosis, so the contribution of NLRP3/pyroptosis in DR needs to be further evaluated to provide new directions for early diagnosis or treatment of DR.

## 5 The activation of NLRP3/pyroptosis in other ophthalmic diseases

NLRP3-mediated pyroptosis is not only present in DR but is also involved in other ophthalmic diseases, for example, NLRP3 inflammasome activation caused by elevated tear osmolarity is the initial signal of corneal inflammation associated with dry eye, and pyroptosis is a prominent result of NLRP3 activation. Zhang et al. observed that hyperosmolarity induced pyroptosis in corneal epithelial cells, and that Calcitriol effectively alleviated damage by inhibiting the NLRP3-ACS- CASP1-GSDMD pathway (184). Chen et al. found that microglia undergo pyroptosis releasing inflammatory cytokines to induce RGC death was associated with glaucomatous vision loss, genetic deletion of the pyroptosis effector GSDMD significantly ameliorated RGCs death and retinal tissue damage in acute glaucoma (181). Particulate matter (PM_2.5_) induced increased oxidative stress and subsequent NLRP3 inflammasome-mediated pyroptosis was observed in trabecular meshwork cells, leading to ocular hypertension and glaucoma, and NAC ameliorated these symptoms (185). In another study, it was similarly observed that PM_2.5_ induced pyroptosis in corneal epithelial cells *via* the ROS/NLRP3/pyroptosis pathway. Activation of NLRP3/pyroptosis was also observed in Candida albicans keratitis, and knockdown of NLRP3 significantly alleviated the pyroptosis and corneal inflammatory response, making it an attractive target for the treatment of fungal keratitis (186). Sun et al. found that MCC950 alleviated Aβ-induced pyroptosis in age-related macular (187). In conclusion, NLRP3, the most studied inflammasome, which induced the pyroptosis in many ocular diseases, therefore targeting the NLRP3/pyroptosis pathway may be a new target for the treatment of many ophthalmic diseases.

## 6 Therapy targeting NLRP3 and pyroptosis in diabetic retinopathy

Diabetic retinopathy is considered to be a chronic low-grade inflammatory disorder, while the function of NLRP3 inflammasome and pyroptosis activation in the pathogenesis of DR is well established. Therefore, small-molecule inhibitors targeting the inflammasome and pyroptosis in DR might improve clinical outcomes. Fortunately, several inhibitors of NLRP3 and pyroptosis have been identified, including direct inhibitors of NLRP3, caspase-1, and GSDMD, as well as indirect inhibitors that target inflammatory components or associated signaling events. However, animal or human DR model testing of some inhibitors of pyroptosis has not been executed. At the same time, some of the inhibitors tested are potentially risky because their precise target of inhibition is not fully understood. In this review, several pyroptosis inhibitors that have been examined have been summarized (**Table 2**). For instance, to prevent pore formation and the discharge of inflammatory mediators, the GSDMD inhibitors NSA and disulfiram covalently bind to Cys191 on the GSDMD. This prevents the pyroptosis of retinal cells (175, 189). MCC950 is a highly specific inhibitor of NLRP3, Zhang et al. explored the anti-inflammatory effects of MCC950 treatment on HRECs after HG stimulation and the potential mechanisms to investigate the role of inflammasome-mediated cell death in DR. They demonstrated that MCC950 treatment significantly attenuated the initiation of the NLRP3 inflammasome in HRECs, which in turn reduced the mRNA expression levels of NLRP3, caspase-1 and proIL-1β in HRECs. More importantly, many studies demonstrated that MCC950 inhibits NLRP3 while significantly suppressing GSDMD expression in RMECs, microglia and Müller cells, thereby inhibiting retinal cell pyroptosis, reducing the rate of retinal vascular leakage and delaying the progression of DR in mice, The above studies provide strong evidence that MCC950 is a very promising drug for DR treatment. Mechanistically, MCC950 greatly protects retinal cells from HG-stimulated dysfunction by inhibiting the binding of NEK7 to the NLRP3 inflammasome (120, 191).

**Table 2:** Inhibitors target NLRP3/pyroptosis.

Inhibitor	Structure	Mechanism	Research in other eye diseases
**Necrosulfonamide (** 175)	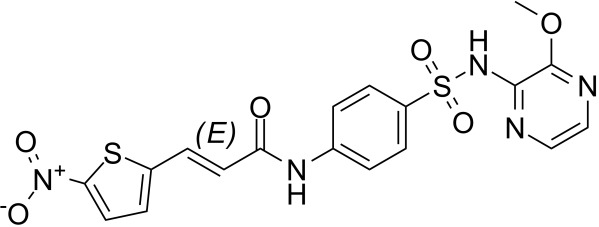	Inhibit the expression of NLRP3 and GSDMD, and reverse the effects of high-glucose on ARPE-19 cell pyroptosis.	Acute keratitis (188)
**Disulfiram** (189)	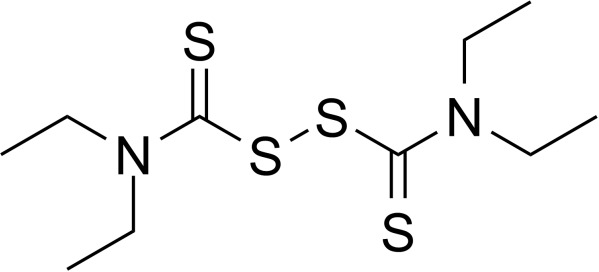	Inhibit pyroptosis by inhibiting the GSDMD pore formation	Dry eye (184) Acute glaucoma (190)
**MCC950** (120, 176, 191)	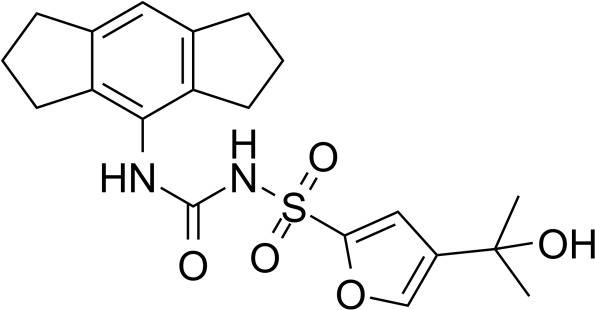	MCC950 blocks canonical and non-canonical NLRP3 activation,	Retinal Neurodegenerative Diseases (176) Age-Related Macular Degeneration (187) Age-Related Macular Degeneration (192) Cataract Formation (193) Lacrimal Glands (194)
**Tranilast** (176)	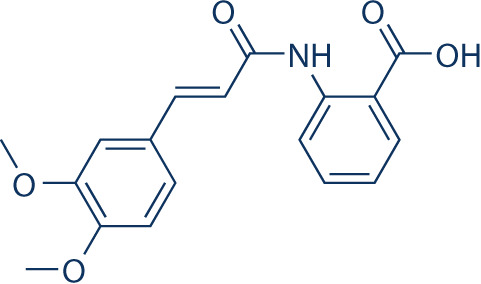	Binds NACHT domain and inhibits the NLRP3 polymerization and activation	Viral infection of the cornea (195) Dry eye (196)
**Tonabersat** (197–199)	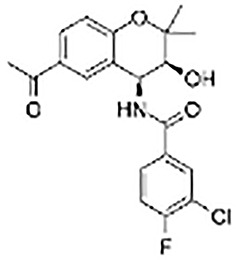	Inhibits Connexin43 Hemichannel Opening and NLRP3 inflammasome activation	Age-Related Macular Degeneration (200) Light-induced retinal degeneration (201)
**EGCG** (161)	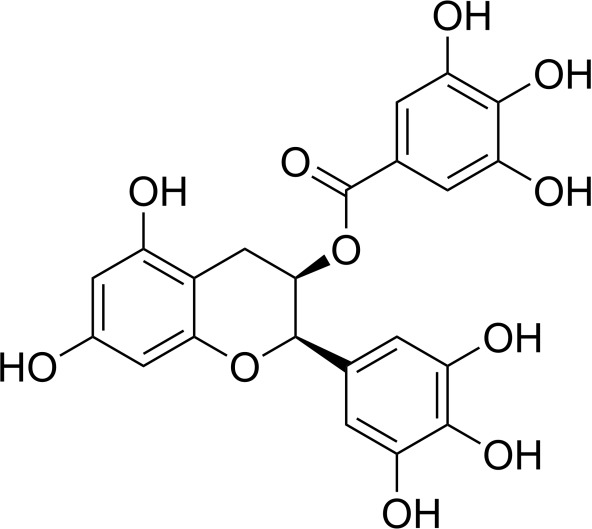	Targeting the ROS/TXNIP/NLRP3 inflammasome axis	Light-induced retinal death (202) Chronic glaucoma (203) Dry Eye (204, 205)
**Resolvin D1** (180)	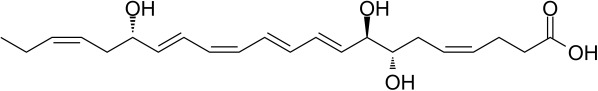	Suppressing the activation of NF-kB and inhibiting the activation of NLRP3	LPS-induced keratitis (188, 206) Allergic eye disease (207) Immunoinflammatory response of the uveitis (208)
**Peptide5** (143, 209, 210)	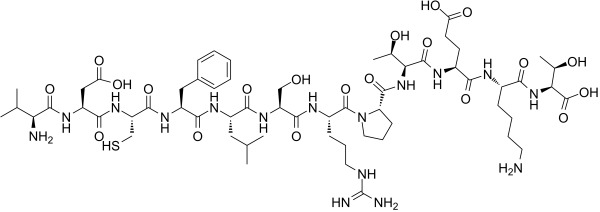	Blocking connexin43 hemichannel and inhibiting the activation of NLRP3	Light-induced retinal inflammation (210) Optic neuropathy (211)
**Hydrogen sulfide** (212, 213)	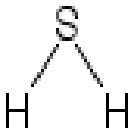	H_2_S protects RPE cells from damage caused by high glucose by inhibiting ROS formation and NLRP3 inflammasome activation	Age-Related Macular Degeneration (214) Glaucoma (215)
**Sulforaphane** (216–218)	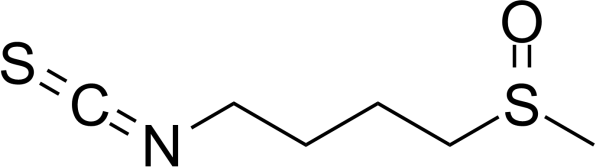	Inhibiting the activation of Nrf2 and NLRP3 inflammasome	Age-Related Macular Degeneration (219) Retinal ischemia-reperfusion (220)
**H3 relaxin** (148)	\	Alleviate pyroptosis through targeting the P2X7R/NLRP3 pathway	Renal inflammatory pyroptosis (221)
**Methylene Blue** (222)	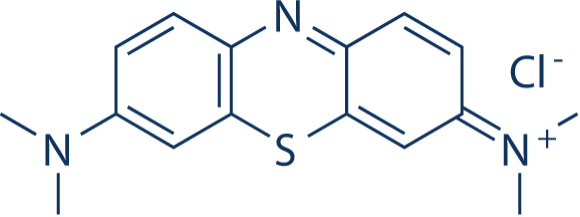	Inhibiting the NF-κB signal pathway	Retinal photoreceptor degeneration (223) Ischemic proliferative retinopathy (224)
**Z-YVAD-FMK (** 169)	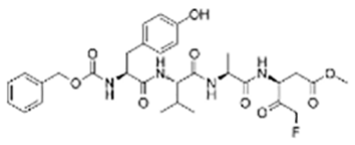	Inhibiting the caspase-1/4	Age-Related Macular Degeneration (225, 226)

NLRP3 NOD-like receptor protein 3, GSDMD gasdermin D, ARPE-19 adult retinal pigment epithelial cell line-19, ROS reactive oxygen species, TXNIP thioredoxin interacting protein, NF-κB nuclear factor-kappa B, Nrf2 Nuclear factor erythroid2-related factor 2, P2X7R purinergic receptor.

Other indirect inhibitors of the inflammasome, such as connexin 43 hemichannel, mediate RPE pyroptosis in DR *via* the ATP/NLRP3 inflammasome pathway, which can be blocked by peptide5 (142) and tonabersat. Mugisho et al. demonstrated that Peptide5 significantly reduced the incidence of DR-induced beading and vessel dilation, decreased the severity of vitreous and retinal hyper-reflective foci, and reduced subretinal fluid accumulation. In addition, Peptide5 resulted in reduced upregulation of connexin43 and GFAP compared to controls, inhibited the upregulation of the inflammatory markers IL-1β/18. Louie et al. further demonstrated a significant reduction in the expression of NLRP3, pro-inflammatory cytokines IL-1/18 and VEGF in Müller cells *via* the hemichannel inhibitor tonabersat. The above studies provide evidence that Peptide5, as well as other Connexin43 hemichannel blockades, can inhibit the inflammatory response and cell death by targeting the upstream signaling of NLRP3 (199). In addition, in DR pathogenesis, high glucose stimulates activation of NLRP3 inflammasome mediated by P2X7R, which could be significantly inhibited by H3 Relaxin. H3 Relaxin is a bioactive peptide with an insulin-like structure, which has been reported to be effective in diabetic cardiomyopathy. In STZ-treated retinas, disorganized membrane discs were observed, mitochondria were degraded, the number of synapses and synaptic vesicles in the inner and outer plexiform layer was reduced, and the ganglion cell layer showed swollen ganglion nuclei and dilated endoplasmic reticulum. After administration of high doses of H3 Relaxin, the above phenomena were significantly improved. In addition, the levels of NLRP3, ASC, caspase-1, IL-18/1β and GSDMD were significantly increased in RMECs after STZ treatment. After administration of H3 Relaxin, the expression of all pyroptosis-related proteins decreased. The above results suggest that HG triggered the activation of P2X7R and subsequently NLRP3 inflammasome in RMECs, while H3 Relaxin significantly reduced HG-induced expression and activation of NLRP3 inflammasome by inhibiting P2X7R, which in turn inhibited pyroptosis (148). Epigallocatechin gallate (EGCG), a major bioactive tea compound, could suppress the ROS/TXNIP/NLRP3 inflammasome pathway in Müllers *via* inhibition of streptozotocin (STZ)-induced DR in a mouse model (161). Garcinia cambogia also prevents the TXNIP/NLRP3 activation *via* reducing the levels of ASC, NLRP3, cleaved-IL-1β, cleaved-caspase-1, and TXNIP (227). Palbinone has a significant effect on attenuating the inflammatory response of the blood vessels and reducing vascular permeability in the retinal of DR by inhibiting NLRP3 activity, and more importantly, RT-PCR and immunofluorescence staining suggest that Palbinone may be a promising pharmacological agent to inhibit pyroptosis-mediated cell death for the treatment of DR (228). Similarly, rhodopsin has been shown to exert antioxidant effects in a mouse model of DR, activating the Nrf2 pathway and inhibiting NLRP3, caspase1, ASC, and cleaved IL-1β levels in Müller cells (218). Hydrogen sulfide could also protect high glucose-induced RPEs death and inflammatory damage from oxidative stress by suppressing the production of ROS and the activation of NLRP3 inflammasome (229). Resolvin D1 treatment in DR rats effectively reduces NLRP3-mediated inflammatory factor secretion in the retinal tissue by inhibiting the NF-kB pathway (180). As the pyroptosis and inflammasome in several ocular diseases are intensively studied and the number of affected individuals concerning inflammatory disorders is increasing, future clinical translation will be facilitated by specific and direct inhibitors of pyroptosis, with a focus on the use of precision medicine in inflammatory diseases.

## 7 Conclusion

Previous studies have found inflammatory responses at all stages of DR and NLRP3 as a causative factor in its pathogenesis. In DR, NLRP3 inflammasome can recognize multiple diabetic metabolic factors as endogenous danger signals, in turn activating CASP1. These diabetic metabolic factors include ATP and cytoarchitectural instability, such as rupturing of the lysosome, dysfunction of the mitochondria, and molecular or ionic perturbations, such as K efflux, ROS, and Ca^2+^ signaling. Activated CASP1 can then cleave GSDMD to C-GSDMD and N-GSDMD; N-GSDMD causes perforation of the cell membrane to form non-selective pores, causing the cells to swell and resulting in pyroptosis. On the other hand, CASP1 cleaves pro-IL-1β/18 to mature IL-1β/18, which are released through GSDMD-induced pores, promoting pyroptosis. However, current research on NLRP3 activation and pyroptosis in DR is still only the tip of the iceberg, and many questions remain unanswered at the molecular mechanism of pyroptosis. For instance, most evidence on pyroptosis is limited to activating the NLRP3 inflammasome, CASP, and other proteins rather than GSDMD activation in Müller cells, pericytes, endothelial cells, and RPE cells. Although evidence indicates the occurrence of high glucose-induced retinal pericyte pyroptosis through the NLRP3-caspase-1-GSDMD pathway, more direct evidence is needed to support the role of GSDMD in retinal cells. Furthermore, although it appears that a variety of cells undergo pyroptosis in DR, it remains unclear which cells undergo pyroptosis first, or whether the interactions between cells undergoing pyroptosis need to be further investigated. Each GSDM family member’s function in various disorders may vary, here, we focused on the role of GSDMD in DR. More research is needed to explore whether other members of the GSDM family are involved in the development of DR. Moreover, in disease models we usually study a single mode of death in isolation, whereas in fact under different pathological conditions the pattern of cell death is dynamic and it is likely that multiple modes of death co-exist. Therefore, it is necessary to look at the mode of cell death as a whole and dynamically observe their role in DR, which may be more relevant for clinical translation and application.

Several promising compounds are currently available that effectively inhibit the onset of pyroptosis, offering promising therapeutic directions for the management and treatment of ocular diseases, including DR. Although we have summarized the therapeutic potential of pyroptosis-related inhibitors in DR, most of the evidence is from cellular or animal studies, and have not yet been validated in clinical trials; therefore, further studies are needed to understand their clinical efficacy. On the other hand, traditional drug delivery by repeated vitreous cavity injections (230, 231) may cause increased intraocular pressure, cataracts, and other complications for the patient (232, 233). Therefore, in addition to investigating effective targeted therapeutic agents, finding better drug delivery methods is necessary to improve the outcome of patients with DR. Recently, gene therapy (183, 234–236) and nanomedicines for pyroptosis have received great attention (237). The findings of this research suggest that utilizing nanomedicine in patients with DR to target the lesion, with the potential to accumulate over time and gradually release, could hold great promise for clinical application and offer new avenues for enhancing clinical outcomes.

## Author contributions

Conception and design of study, XZ, JW and GT. Drafting of article, XZ, JW and GT. Revision of draft, XZ and GT. All authors contributed to the article and approved the submitted version.
